# Higher Loading of Gold Nanoparticles in PAD Mesenchymal-like Stromal Cells Leads to a Decreased Exocytosis

**DOI:** 10.3390/cells11152323

**Published:** 2022-07-28

**Authors:** Jennifer Oberländer, Rafael Ayerbe, Joan Cabellos, Richard da Costa Marques, Bin Li, Nazende Günday-Türeli, Akif Emre Türeli, Racheli Ofir, Eliran Ish Shalom, Volker Mailänder

**Affiliations:** 1 Max-Planck-Institute for Polymer Research, Ackermannweg 10, 55122 Mainz, Germany; oberlaenderj@mpip-mainz.mpg.de (J.O.); dacostamarques@mpip-mainz.mpg.de (R.d.C.M.); 2 Department of Dermatology, University Medical Center of the Johannes Gutenberg-University Mainz, Langenbeckstraße 1, 55131 Mainz, Germany; 3 LEITAT Technological Center, c/Innovació, 2, 08225 Terrassa, Spain; rayerbe@leitat.org (R.A.); jcabellos@leitat.org (J.C.); 4 MyBiotech GmbH, Industriestraße 1 B, 66802 Überherrn, Germany; b.li@mybiotech.de (B.L.); n.guenday-tuereli@mybiotech.de (N.G.-T.); e.tuereli@mybiotech.de (A.E.T.); 5 Pluristem Therapeutics Inc., Matam Park, Building 05, Haifa 3508409, Israel; racheli@pluristem.com (R.O.); ishshalom@pluristem.com (E.I.S.)

**Keywords:** cell therapy, gold nanoparticles, cell labeling

## Abstract

Cell therapy is an important new method in medicine and is being used for the treatment of an increasing number of diseases. The challenge here is the precise tracking of cells in the body and their visualization. One method to visualize cells more easily with current methods is their labeling with nanoparticles before injection. However, for a safe and sufficient cell labeling, the nanoparticles need to remain in the cell and not be exocytosed. Here, we test a glucose-PEG-coated gold nanoparticle for the use of such a cell labeling. To this end, we investigated the nanoparticle exocytosis behavior from PLX-PAD cells, a cell type currently in clinical trials as a potential therapeutic agent. We showed that the amount of exocytosed gold from the cells was influenced by the uptake time and loading amount. This observation will facilitate the safe labeling of cells with nanoparticles in the future and contribute to stem cell therapy research.

## 1. Introduction

The idea of using cells as a therapeutic agent for various diseases has widely expanded over the past years [1]. However, the therapeutic mechanisms and the distribution of the cells in the organism often remain unclear. As methods for tracking cells in the human body are very limited, imaging and contrast agents like nanoparticles have been developed to increase the visibility of the cells in vivo [2,3,4]. To date, cell tracking is often performed using either radionuclides for scintigraphy, PET (positron emission tomography), and SPECT (single photon emission computer tomography), or using superparamagnetic iron oxide nanoparticles for MRI (magnetic resonance imaging) [5,6,7]. Radionuclide imaging has the main advantage of high sensitivity and very small amounts of label can be detected. However, radionuclides with short half-lives make this method very expensive and make long-term tracking impossible [6]. On the other hand, MRI does not use ionizing radiation and tissue can be well visualized. Nevertheless, this method is also relatively cost-intensive and the acquisition time is slow [6,7]. Therefore, there is increasing effort to develop cell tracking markers for CT (computed tomography). These have the benefit that the technology is cost-effective with a fast temporal resolution [7]. Thereby, gold nanoparticles or gold-iron nanoparticles have proven to be very promising tracking tools. Gold features strong contrast properties for CT imaging and, in combination with iron for MRI, a wide range of analysis methods can be covered when combining both materials [3]. Furthermore, gold nanoparticles are already very well studied materials of low toxicity to cells, making them even more promising as a contrast agent for cell therapy labeling [8,9,10]. However, for good CT tracking, a high amount of gold nanoparticles need to be taken up by the cells and, for long-term tracking, the nanoparticles need to remain stable in the cells [11]. While high loading of cells could previously be achieved, the reduction of the exocytosis of gold nanoparticles from cells still remains a challenge [11,12,13].

One type of cell that is currently considered as a potential therapeutic agent against different diseases is the PLX-PAD cell [14,15,16,17,18,19,20,21,22,23,24]. PLX-PAD cells are a cell therapy product under development containing placental expanded (PLX) placenta-derived mesenchymal-like adherent stromal cells [16,21,25]. It has previously been shown that these cells secrete relevant factors as a response to muscle trauma or inflammation to trigger the natural repair mechanisms of the body [16]. Therefore, these cells are currently used in different clinical studies for the treatment of injured muscle.

For a combination of particles and cells to be effective as a therapeutic and imaging reagent, the particles must remain stable in the cells. One factor that can influence the uptake in cells is the so-called protein corona. Furthermore, surface modifications and the size and shape of the particles—among other things—can affect the uptake and exocytosis rate of particles [26,27,28]. When nanoparticles come into contact with biological fluids, proteins directly attach to the surface of the particles and form a protein corona. Previously, it has been shown that the protein corona can alter the uptake of particles into cells, but it can also influence the exocytosis rate of particles from cells [28,29]. To use the nanoparticles as a reliable imaging reagent, it is crucial to keep the exocytosis rate as low as possible to allow long-term tracking of the cells. Besides influencing the uptake into cells, changes in the protein corona composition can also be used to learn about the intracellular pathway followed by the nanoparticles. The type of proteins bound in the corona, therefore, reflects a fingerprint of the intracellular pathway taken by the nanoparticles [30,31]. This intracellular protein corona can be used to predict whether particles are more likely to be exocytosed or to remain inside the cell.

In this study, we analyzed the exocytosis rate of glucose-coated and PEGylated gold nanoparticles from loaded PLX-PAD cells. As the combination of cells and particles should be used in the future for long-term tracking of cells in the human body, it is important to reach a high loading efficiency together with a low exocytosis rate of the GNPs from the cells. Here, we tested two different in vitro loading protocols—one with a lower loading efficiency and another with a higher loading efficiency of GNPs. Afterwards, we analyzed the amount of exocytosed gold and characterized the intracellular protein corona to predict which protocol reached a more stable loading of nanoparticles in the cells.

## 2. Materials and Methods

### 2.1. Synthesis of GNPs

PEGylated and glucose coated gold nanoparticles (GNPs) were synthesized according to previously described methods in a three-step process [3]. A detailed synthesis procedure is given in ESI.

### 2.2. Characterization of GNPs

After the synthesis, the gold concentration of the nanoparticles was determined by ICP-OES or ICP-MS. The size of the particles was determined via multi-angle DLS and TEM [32]. Detailed information on the characterization methods used is given in ESI.

### 2.3. Production and Cell Culture of PLX-PAD Cells

PLX-PAD cells were produced by Pluristem Therapeutics, Ltd. (Haifa, Israel). As previously described [14,25], details about the production and cell cultivation are given in ESI.

### 2.4. Uptake and Exocytosis

Two different protocols for the uptake of GNPs were tested. For both protocols, the PLX-PAD cells were seeded at a density of 500,000 cells per well in a six-well plate (Greiner, Pleidelsheim, Germany) and incubated overnight. All incubation steps were performed in a humidified incubator (37 °C, 5% CO_2_). Afterwards, the cells were incubated with a nanoparticle solution of 300 µg/mL in isotone NaCl (B.Braun, Melsungen, Germany) for 30 min (low loading protocol) or with 300 µg/mL nanoparticles in DMEM (Thermo Fisher Scientific, Waltham, MA, USA) with 10% FBS for 24 h (high loading protocol). After the incubation time, the cells were washed twice with PBS (Thermo Fisher Scientific, Waltham, MA, USA) and then either incubated again in DMEM with 10% FBS for exocytosis measurements or harvested with Trypsin-EDTA (Thermo Fisher Scientific, Waltham, MA, USA) for uptake measurements.

The exocytosis of the GNPs was also determined using two different protocols. Either the collected DMEM was not replaced or the DMEM volume collected was replaced. When keeping the same supernatant during the entire time, the DMEM and the corresponding cells were collected after 2, 6, 24, or 48 h (without supernatant replacement). For the second protocol with supernatant replacement, the supernatant was exchanged with fresh supernatant after collecting it from the cells at the same time point as for the protocol without supernatant replacement. Here, the cells and the supernatant were harvested for analysis as well.

### 2.5. ICP-OES Analysis

Before the analysis of the gold content in the cells and in the supernatant, the cells were digested. Therefore, after trypsinization, cells were centrifuged (300× *g*, 5 min) and the supernatant was discarded. Afterwards, cells were diluted with 1 mL of aqua regia (3:1 hydrochloric acid/nitric acid) and incubated at room temperature overnight on an orbital shaker (300 rpm) followed by dilution with MiliQ water to 10 mL before measurement. The cell culture supernatants for the determination of exocytosed gold content were collected from the cells and treated with aqua regia followed by incubation and dilution to 10 mL with water. The gold concentration was determined by ICP-OES (SpectroGreen, Spectro/Ametek, Kleve, Germany). The calibration curve was prepared using 0.1, 0.5, 1, 5, and 20 ppm gold standard solution (stock 1000 mg/L Au TraceCERT^®^, Sigma Aldrich, St. Louis, MO, USA). Untreated cells and cell culture media were used to deduct background signals. The amount of gold was calculated using the concentration resulting from the ICP OES and the volume of the sample.

### 2.6. ICP-MS Analysis

ICP-MS analysis was used to determine the cell loading efficiency. After incubation of PLX-PAD cells with GNPs, incubation cell culture media, washing media, and GNP exposed cells were digested in 1 mL aqua regia (3:1 hydrochloric acid/nitric acid). The obtained digestion solutions were properly diluted to be analyzed by ICP-MS (7500, Agilent, Santa Clara, CA, USA) in duplicates. The quantification was carried out by interpolation in a standard curve obtained from a commercial 1000 ppm gold standard (Inorganic Ventures, INYCOM, Zaragoza, Spain).

### 2.7. Cell Viability Evaluation

The viability of PLX-PAD cells exposed to GNPs was measured with the Alamar Blue™ (Thermo Fisher Scientific, Waltham, MA, USA) assay. PLX-PAD cells were seeded at a density of 10,000 cells per well in 96-well plates and grown in a humidified incubator (37 °C, 5% CO_2_) for 24 h. GNPs were diluted in DMEM with FBS at different concentrations (25, 50, 100, 200, 400, 800, and 1200 µg/mL) and incubated in the incubator with the cells for 24 h. After cell exposure, the cell media was removed and wells were washed twice with PBS. Then, cells were exposed to Alamar Blue™ reagent for 1 to 3 h. After the incubation time, an aliquot of the supernatant was transferred into a new plate and the fluorescence (530/590 nm) was measured with a microplate fluorometer. The fluorescence signal of untreated cells was set as 100% and the viability was calculated as a function of this.

### 2.8. TEM

For the imaging of the cell uptake with the high loading protocol, PLX-PAD cells were washed with PBS after the incubation period and fixed with glutaraldehyde (2.5%) for 1 h. The cells were detached from the Petri dish by scraping and afterwards centrifuged (4 °C, 1000× *g*, 5 min) to obtain a compact pellet. Detailed information about the freezing protocol for TEM imaging is given in ESI.

To observe the internalization and exocytosis of the GNPs for the low loading protocol, the cells were seeded at a density of 50,000 cells per well in 24-well plates and incubated overnight. Before seeding, the plates were equipped with 3 mm plasma-sterilized sapphire discs (M. Wohlwend GmbH, Sennwald, Switzerland) covered with a 20 nm carbon layer. Incubation of the cells with the GNPs was performed for the exocytosis protocol without supernatant replacement to image intracellular localization of the GNPs over time. After the incubation period, the GNPs were removed, the cells were washed with PBS, and the cells were further processed for TEM imaging (described in ESI).

### 2.9. Intracellular Trafficking

For the intracellular trafficking of the GNPs, PLX-PAD cells were seeded at a density of 400,000 cells per well in six-well plates. On the next day, incubation with GNPs was carried out at a GNP concentration of 300 µg/mL for 4 and 24 h. Additionally, a pulse-chase experiment was performed where the PLX-PAD cells were incubated with the GNPs for 4 h followed by an exchange to fresh cell culture medium and a subsequent 20 h incubation. After the incubation time, cells were harvested with Trypsin-EDTA (Thermo Fisher Scientific, Waltham, MA, USA). For each condition, three wells were pooled after harvesting to reach enough intracellular GNPs. PLX-PAD cells were centrifuged (300× *g*, 5 min) and the supernatant was discarded. For cell lysis, cells were suspended in PBS with protease inhibitor (Thermo Fisher Scientific) and EDTA at 4 °C. Cell lysis was carried out through sonication (QSonica Q800R3, QSonica, Newton, CT, USA) at an amplitude of 70% and pulsation frequency of 30 s. Sonication was performed in total for 7 min at 4 °C. Cell debris and nuclei were removed by centrifugation (1000× *g*, 4 °C, 10 min) and, afterwards, the GNPs were collected by centrifugation (5000× *g*, 4 °C, 30 min). To remove unbound and loosely bound proteins, the nanoparticles were washed three times with PBS containing protease inhibitor and EDTA. Afterwards, the hard corona proteins were detached from the nanoparticles using 2% (*w*/*v*) SDS and 62.5 mm Tris-HCl, as previously described by our group [33,34]. Proteins and nanoparticles were separated via centrifugation (5000× *g*, 4 °C, 30 min) and protein-containing supernatant was used for protein quantification, SDS PAGE, and LC-MS analysis.

### 2.10. Protein Quantification and SDS-PAGE

Proteins were quantified by the Pierce 660 nm Protein Assay (Thermo Fisher Scientific) according to the manufacturer’s manual. The adsorption was measured with a Tecan Infinite M1000 plate reader using bovine serum albumin (Merck, Kenilworth, NJ, USA) for a calibration curve.

After quantification, the proteins were separated with SDS-PAGE and stained by Silver Staining according to the manufacturer’s recommendations. A total of 2 µg protein in 26 µL total volume was mixed with 4 µL NuPage Reducing Agent and 10 mL of NuPage LDS Sample Buffer (Thermo Fisher Scientific). The sample was loaded on a 10% Bis-Tris-Protein Gel using SeeBlue Plus2 Pre-Stained (Invitrogen, Waltham, MA, USA) as a molecular marker and NuPage MES SDS Running Buffer. Gels were stained with Pierce Silver Stain Kit (Thermo Fisher Scientific) according to the manufacturer’s protocol.

### 2.11. In-Solution Digestion, Liquid Chromatography-Mass Spectrometry (LC-MS), and Protein Identification

The in-solution digestion, liquid chromatography-mass spectrometry, and protein identification were performed as previously described by our group with slight modifications [33,34,35]. Detailed information about the digestion and LC-MS analysis is given in ESI [36,37].

### 2.12. Protein Annotation

After protein identification, we identified all corona proteins with an enrichment of 1.5-fold compared with the lysate proteins. The enriched corona proteins were forwarded to an analysis with the functional annotation tool DAVID (Version 6.8, https://david.ncifcrf.gov/home.jsp accessed on 15 November 2021) [38,39]. The enriched proteins were analyzed by functional annotation clustering, implementing the database for GOTERM_CC_FAT with medium classification stringency to consider the cellular compartments. The following GOTERMS were considered for the study: extracellular vesicle, cell surface, secretory vesicle, endoplasmatic reticulum, early endosome, cytoplasmic region, endocytic vesicle, and nucleosome.

## 3. Results and Discussion

**Figure 1:**
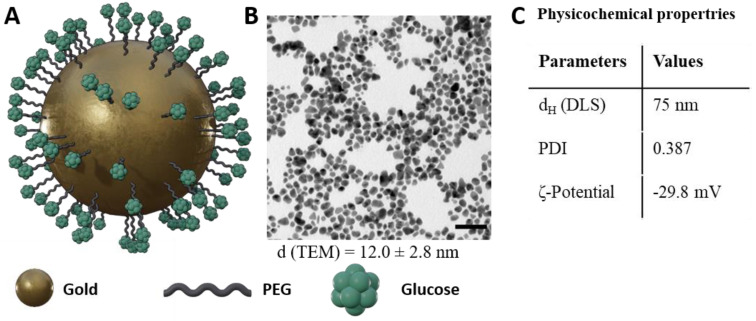
(**A**) Schematic representation of PEGylated and glucose-coated gold nanoparticles. (**B**) Representative TEM images of GNPs. The size of the nanoparticles is given in nm ± SD. Scale bar = 50 nm. (**C**) Physicochemical properties of the GNPs.

For the purpose of evaluating the influence of the loading amount on nanoparticles on their exocytosis, glucose-PEG-coated gold nanoparticles (GNPs) were synthesized. In this study, we focused on one type of nanoparticle to demonstrate changes in the nanoparticle exocytosis behavior independently from particle size or surface functionalization. Gold nanoparticles were chosen as a widely used type of nanoparticle and as a particle type that could be used in the future as an imaging reagent for stem cells. To achieve a higher stability of the nanoparticles under physiological conditions, the GNPs were surrounded with HS-PEG-COOH via gold–thiol interactions. Carboxylic functionalization of PEG was then used for coupling of glucose on the nanoparticles to achieve an increased uptake of the GNPs into cells [2]. In Figure 1, an illustration of the surface functionalization of the nanoparticles can be seen, as well as a representative transmission electron microscopy (TEM) picture. TEM showed an average size of the gold nanoparticles of 12.0 ± 2.8 nm. Dynamic light scattering (DLS) revealed a significantly larger size of the GNPs of 75 nm (Figure S1). However, using TEM, only the size of the gold nanoparticle core is measured (i.e., without the PEG and glucose coating contributing to the size measurement). In contrast in DLS, where the whole hydrodynamic diameter of the particles is measured. As the PEGylated and glucose-coated nanoparticles show a negative ζ-potential, the particles can, in addition, build up more electrostatic interactions in liquid dispersion, and thus show a larger diameter size than that measured by TEM images.

To evaluate the exocytosis of the GNPs from PLX-PAD cells, we used two different uptake and exocytosis protocols. For a lower loading of nanoparticles in the cells, we used a protocol with a short incubation time (30 min). However, to still have a sufficient uptake of nanoparticles in cells for this protocol, we incubated the cells without any proteins in isotone NaCl solution, as it was previously shown that the protein corona that formed in cell culture media with FBS reduces the uptake of nanoparticles into cells [40]. This phenomenon, known as the *stealth effect*, was intended to be avoided in this protocol by incubating the cells without the presence of proteins in the exposure media [29]. In the second protocol, we incubated the GNPs with the PLX-PAD cells for 24 h in DMEM with FBS. There, we expected to have a higher uptake and intracellular internalization of the nanoparticles because of the longer incubation time, despite the presence of proteins. The FBS was added to the cell culture media to avoid potential damage or change in cell functions due to the long incubation time without proteins.

Determination of the gold concentration in the cells was carried out using ICP-OES and ICP-MS. ICP-OES is mainly used to determine the concentration of metals in complex solutions. For the analysis, the samples are first atomized and ionized in an argon plasma. Then, the ions and atoms are excited and emit electromagnetic radiation, which can be assigned to the different elements as discrete lines and by that quantified [41]. In ICP-MS, ions are extracted from the plasma and separated on the basis of their mass-to-charge ratio in order to quantify the concentration. For the injection of the samples into the plasma, the samples are sprayed into a nebulizer in order to form small droplets. As cells and cell clumps in the sample could result in an inhomogeneous solution and lead to poor aerosol formation, the cells were previously dissolved overnight in aqua regia. Afterwards, the gold concentration in the cell solutions was determined for both uptake protocols and exocytosis protocols.

**Figure 2:**
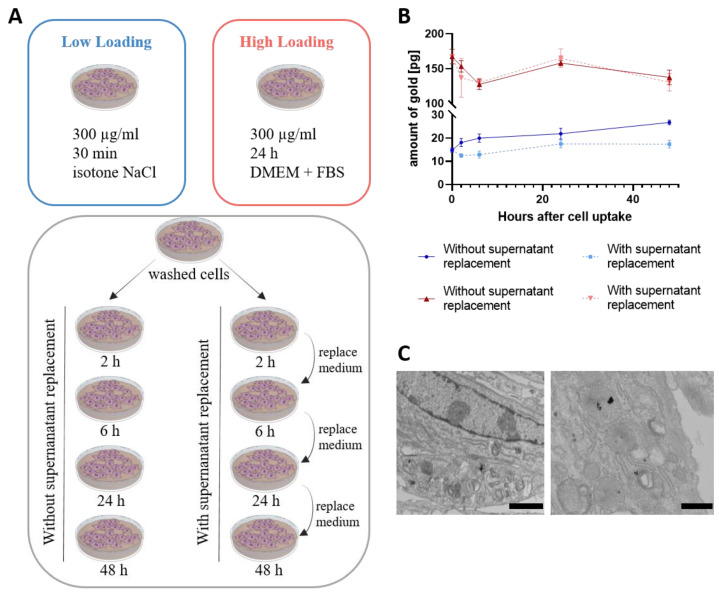
(**A**) Schematic representation of the different incubation protocols used for incubation. PLX-PAD cells were incubated either for 30 min with 300 µg/mL GNPs in isotone NaCl solution (blue) or for 24 h with 300 µg/mL GNPs in DMEM with FBS (red). After uptake of GNPs, the cells were further incubated in DMEM with FBS for up to 48 h either with regular exchange of the medium (grey box right) or kept in the same medium (grey box left). (**B**) Calculated gold concentration per cell. Cells were harvested after incubation with GNPs and after incubation with DMEM, respectively. Afterwards, cells were analyzed with ICP-OES and the amount of gold per cell was calculated. Mean values ± SD. *n* = 3. (**C**) Representative TEM images of PLX-PAD cells after uptake of 300 µg/mL GNPs in DMEM + FBS. Images show the internalization of the GNPs in the PLX-PAD cells. Left scale bar represents 2 µm, right scale bar 500 nm.

In Figure 2A, a schematic representation of the different protocols used for the incubation can be seen. Figure 2B shows that the different incubation protocols indeed resulted in different amounts of gold in the cell suspension, suggesting that the uptake of GNPs was higher for the high loading protocol than for the low loading protocol. It can be seen at time point 0, which displays the measurement directly after uptake of the nanoparticles, that the amount of gold in the high loading protocol was 165 pg per cell, while for the low loading protocol, it was 15 pg per cell. Therefore, the high loading protocol led to a particle amount 10 times higher in the cell than the low loading protocol. Additionally, the recovery rate of the gold in comparison with the incubation solution was determined. Therefore, the amount of gold was analyzed in the supernatant of the washing steps and in the cells via ICP-MS. Here, we showed that, for the low loading protocol, only 6.4% of the available gold from the incubation solution was taken up in the cells (Table S1). On the other hand, with the high loading protocol, 27.9% of the gold was taken up by the cells. As longer incubation protocols and longer exposures to nanoparticles could cause damage or toxicity to cells [42], we determined the viability of the PLX-PAD cells with different concentrations of the GNPs (Figure S2). Thereby we demonstrated that the particles do not have toxic effects on the cells and nearly 100% of the cells were viable after a long incubation.

After uptake of the nanoparticles, the cells were incubated again with DMEM for analysis of the exocytosis of the nanoparticles. First, we determined the amount of gold in the cells when the cell culture media was kept the same the whole time (Figure 2A, grey box left). However, what the behavior might be in vivo should always be considered. There, exocytosed particles would be removed relatively quickly by other cells or body fluids. Thus, it is unlikely that exocytosed particles reabsorb on the cells and are taken up again. The amount of gold in the cells was also analyzed after replacing the supernatant regularly (Figure 2A grey box right). In Figure 2B, it can be seen that the intracellular gold concentration does not increase or decrease drastically for both protocols. However, looking deeper into the results, it can be seen that the amount of gold increases slightly with time for the low loading protocol both without and with supernatant replacement. This indicates that, after the uptake time, particles could be still attached to the surface of the cells and were available to be taken up with further incubation time. By washing and harvesting for ICP-OES analysis, particles that are attached to the surface might be lost. With supernatant replacement, this increase stopped after 24 h and the intracellular gold concentration leveled off at a gold amount of 17 pg per cell. Without supernatant replacement, the gold amount increases up to 26 pg per cell, which may indicate a further uptake either of exocytosed GNPs or GNPs from the cell surface. For the high loading protocol, no difference could be observed with or without supernatant replacement. Here, a small decrease in the gold amount can be seen after 2 and 6 h. This could be due to a release or exocytosis of nanoparticles from the cells. However, after 24 h, the gold amount increases to the same level as before and decreases afterwards again slightly to 130 pg per cell. The increase in the gold amount could also be due to an uptake of GNPs that were still attached to the surface and reuptake of exocytosed GNPs occurred. Because, in TEM pictures (Figure 2C), no GNPs could be observed on the cell surface with the high loading protocol, uptake of attached particles on the surface seems to be improbable and reuptake of exocytosed GNPs seems to be more likely. Therefore, the amount of exocytosed gold in the supernatant of the cell was further analyzed.

**Figure 3:**
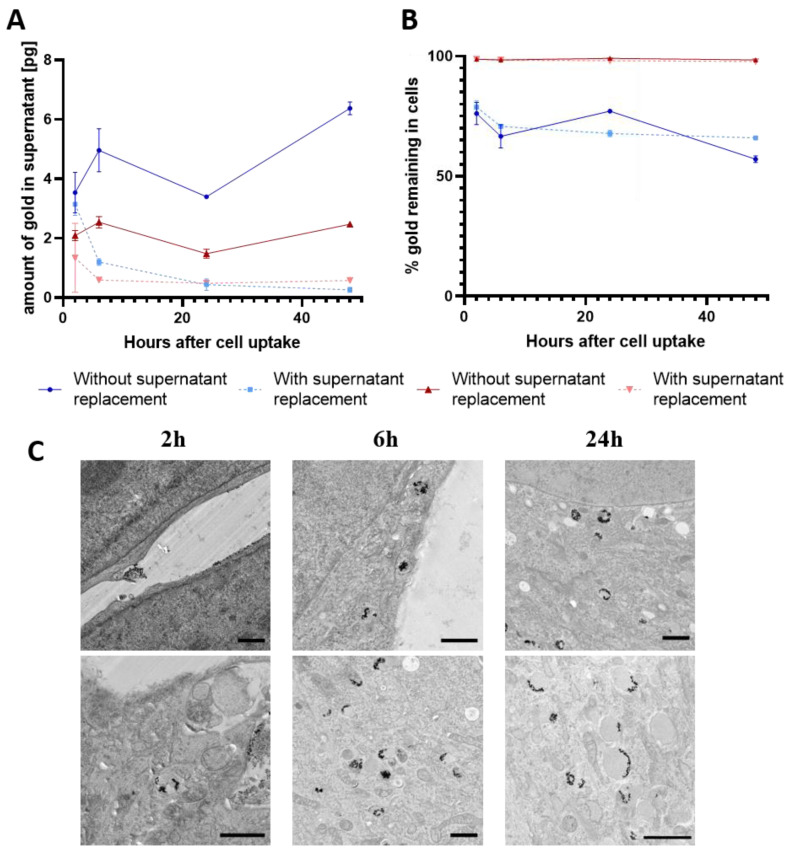
(**A**) Calculated amount of gold in supernatant determined by ICP-OES after incubation of PLX-PAD cells with GNPs and further incubation with cell culture media. The low loading protocol is shown in blue and the high loading protocol in red. The amount of gold is given in pg exocytosed per cell as mean ± SD. *n* = 3. (**B**) Calculated gold amount remaining in PLX-PAD cells after allowing for exocytosis given in percent. Values are displayed as mean ± SD. *n* = 3. (**C**) Representative TEM images of PLX-PAD cells after a first incubation with low loading protocol followed by a second incubation (2 h, 6 h, or 24 h) in cell culture media allowing for internalization and exocytosis. Scale bars are 1 µm.

To further determine the exocytosis of the GNPs from PLX-PAD cells, we analyzed the amount of gold in cell culture media after allowing exocytosis from the cells. In Figure 3A, the calculated exocytosed gold concentration per cell can be seen. To facilitate a comparison between intracellular gold concentration and that in the supernatant, the amount of gold was also given per cell here. Considering the samples without supernatant replacement, it is noticeable that the amount of gold in the supernatant increased for both protocols (low and high loading) until 6 h, then decreased up to 24 h before it increased again up to 48 h. As the supernatant was kept and harvested at the recommended time point, it is very probable that the particles, which were previously released from the cell, were taken up again. Looking at the intracellular gold concentration, an increase in the concentration for the high loading protocol could be observed, which supports the re-uptake theory when the particles are not removed from the cell culture media. This has also been observed previously for gold particles [26]. There, after a short time, a plateau was reached where exocytosis and re-uptake were in equilibrium [26]. However, under in vivo conditions, it is more probable that the exocytosed particles are removed from the surrounding area of cells by other cells (i.e., phagocytic cells). It is also important to pay attention to additional physiological dynamic removal processes that can occur. Considering this for the protocol, we observed that, with regular supernatant replacement, the amount of exocytosed gold decreased over time. Most of the gold was measured in cell culture media after 2 h and decreased rapidly to 6 h. After 24 h and 48 h, only a small amount of gold was measured in the supernatant. Therefore, in accordance with previous studies on different cell types, the exocytosis of nanoparticles seems to be fast, but decreases with time [26].

To allow a better comparison of the exocytosis rate between the different loading protocols, the percent of gold remaining in the cells was calculated (Figure 3B). Therefore, the amount of gold quantified after the uptake of the particles and the amount quantified in the supernatant was used for calculation. While 98% of the gold remained in the cells for the high loading protocol, only 80% remained after 2 h and 66% remained after 48 h for the low loading protocol. Particles could either be released from the cell surface or excreted from the cells. A higher loading of the GNPs in the PLX-PAD cells (by means of a more prolonged incubation protocol) also seems to reduce the exocytosis of the loaded particles from the cells. This can be seen by consideration of the total amount of gold exocytosed and by looking at the amount of exocytosed gold given in percent. These observations could also be confirmed in the TEM images (Figure 3C and Figures S4–S6). To visualize internalization, TEM images were made for the low loading protocol after uptake and additional incubation in cell culture media for 2, 6, and 24 h. While, after 2 h, a high number of particles could be still seen on the cell surface (Figure S4), after 24 h, nearly no more particles attached to the cell surface were observed. In addition, after 6 and 24 h, increasingly more particles seemed to be located in lysosomes, which suggests that the particles were internalized into the cells. Previously, Yu et al. synthesized temperature-responsive GNPs for long-term tracking of stem cells. Here, by adjusting the temperature, an increased uptake and a reduced exocytosis could be observed. However, still, a non-negligible amount of GNPs was secreted within 24 h, which could lead in vivo to a loss of signal or unspecific signal from other cells [11]. During other studies, GNPs were coated using poly-l-lysin or PEG coupled to a trans-activator of transcription peptide to increase the uptake of GNPs in stem cells, and thereby increase the CT signal. However, increased uptake did not lead to a decreased exocytosis of GNPs, which makes long-term tracking still problematic [12,13]. Using our glucose-PEG-coated GNPs and adapting the incubation protocol, the exocytosis rate was significantly reduced and a high loading of the stem cells could still be achieved. This could be used in the future for better long-term tracking of stem cells.

**Figure 4:**
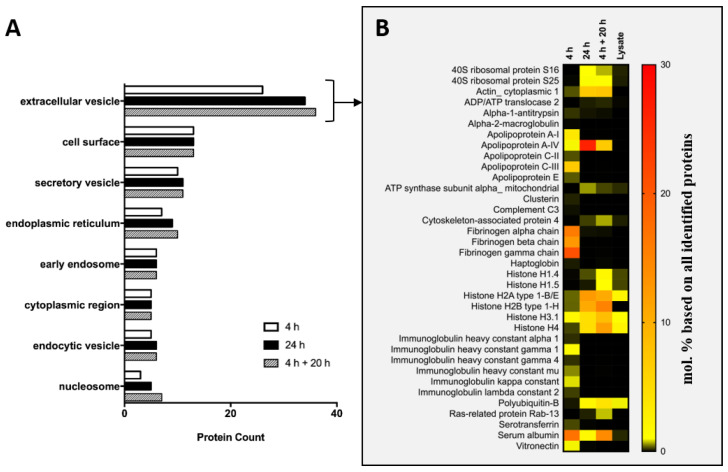
(**A**) Annotation of the proteins bound on the particles to intracellular compartments. Proteins were desorbed from GNPs after incubation and lysis of the PLX-PAD cells. The protein corona was analyzed by LC-MS and proteins assigned to selected intracellular compartments using DAVID-based functional annotation clustering by GOTERM_CC_FAT. (**B**) Heatmap of all proteins assigned to the extracellular vesicle GOTERM. Different incubation time points are displayed in comparison with cell lysate of PLX PAD cells. A detailed list of all proteins found in the protein corona and annotation to the intracellular GOTERMs can be found in ESI and in a supplemented Excel sheet.

The next step was to analyze whether proteins attached to the surface of the GNPs indicate if particles stay intracellular or if they are exocytosed. Therefore, we incubated the particles with the PLX-PAD cells, harvested and lysed them, and collected the proteins in the protein corona around the GNPs. The protein corona develops dependently on proteomic milieu around the particle. Therefore, with this method, it is possible to determine the pathway to which the GNPs belong [30,31]. As it is necessary for this analysis that the nanoparticles were internalized into the cell and were no longer on the surface, the protocol was adapted. Instead of an incubation of 30 min in isotone NaCl solution, the GNPs were incubated for the low loading protocol for 4 h in DMEM with FBS at the same concentration. Therefore, we ensured that we still have a lower and shorter loading of GNPs in the cells, but reach a sufficient amount of particles in the cells to isolate them in the protein corona analysis. Additionally, a 4 + 20 h condition was analyzed to ensure internalization and processing for a possible exocytosis of the GNPs. The proteins were visualized via SDS-PAGE (Figure S3) and analyzed via LC-MS (Figure 4 and Figure S7). A detailed list of all identified proteins is provided in an excel file as supplementary material. No clear differences between the conditions via SDS PAGE were observed. However, a clear distinction between the protein corona composition of the GNPs and the lysate of the PLX-PAD was noticed. This difference confirms that the protein corona of the GNPs was efficiently isolated from the cell lysate.

After the analysis of the SDS-PAGE, the protein corona was additionally analyzed via LC-MS. A bottom-up proteomic approach was used for this protein analysis [43]. Therefore, proteins were desorbed from nanoparticles followed by digestion into peptides with trypsin. Peptides were separated by liquid chromatography and ionized by electrospray ionization. In the mass spectrometer, the mass-to-charge ratio of the ionized peptides was recorded. With the use of a deconvolution software and protein databases, these signals can be assigned to specific proteins, and through this, the composition of protein mixtures can be determined [43]. Here, we used this proteomic approach to analyze the intracellular fate of the GNPs. First, we considered the TOP 10 proteins in the protein corona for all conditions. Thereby, it can be seen that the TOP proteins binding on the GNPs after 4 h differ from the proteins binding after 24 h and 4 + 20 h. After 4 h incubation, serum proteins, namely fibrinogen and serum albumin, were predominantly measured. After more prolonged incubation, this protein adsorption pattern changed towards intracellular proteins e.g., histones and actin.

Next, we analyzed to which intracellular compartments the proteins in the protein corona belong. Therefore, the enrichment factor of the proteins in the protein corona compared with cell lysate was calculated. Only proteins with a 1.5-fold enrichment were considered for annotation to an intracellular compartment. First, we used DAVID to assign the number of the enriched proteins of each condition to the different cellular compartments (Figure 4A). We observed that most of the enriched proteins of all conditions are associated with extracellular vesicles. The highest protein count for extracellular vesicle proteins can be seen in the 4 + 20 h condition and the least protein count in the 4 h condition. For the categories—cell surface, secretory vesicles, early endosomes, cytoplasmic region, and endocytic vesicles—no difference between the different conditions was observed. The higher protein count for the 4 + 20 h condition comes from the fact that, in total, more proteins were enriched in the corona for this condition, indicating that these particles traversed longer through the cells and encountered a more complex protein milieu.

In order to determine whether the protein corona indicates if a particle is exocytosed or not, we analyzed the proteins annotated to the extracellular pathway (Figure 4B). Here, again, the proteins found in the protein corona after 4 h of incubation clearly differ from the proteins in the corona after 24 h and 4 + 20 h. Many proteins that were already found under the TOP 20 list were observed here again, but some proteins that are not that highly represented could also be annotated to this group. After 4 h of incubation time, more apolipoproteins and vitronectin could be seen in the protein corona. However, after further incubation (i.e., 24 h and 20 + 4 h), the protein composition changed and the amount of intracellular proteins increased. Moreover, only one representative of the proteins involved in the exocytosis, namely Rab-13, could be detected in a small amount in the protein corona after 24 h. However, Rab-13 is involved in the formation of intracellular vesicles and involved in both endocytotic and exocytotic processes, and cannot be unequivocally assigned as an exocytosis marker [44,45]. The fast exocytosis of the GNPs observed for the low loading protocols is probably due to the protein corona, which was mainly formed by extracellular proteins. The obtained results suggest that prolonged nanoparticle incubation protocols allow intracellular protein corona changes and lead to a deeper and longer internalization in the cells.

## 4. Conclusions

Here, we demonstrated that a longer incubation protocol allows for higher loading of gold nanoparticles in PLX-PAD cells and leads to reduced exocytosis of these nanoparticles from the cells. Moreover, with the analysis of the protein corona, we showed that, with a prolonged incubation time, the serum proteins in the protein corona are exchanged to intracellular proteins, which probably leads to the reduced exocytosis. A low exocytosis rate of gold nanoparticles is absolutely necessary for long-term tracking of stem cells in vivo as background signals from other cells are reduced and the signal intensity of the stem cells remains stable over time. These findings could help to better understand the in vivo fate of stem cells and intracellular biokinetics of glucose-PEG-coated gold nanoparticles. It can also support on the design of nanoparticles’ uptake protocols for the labelling and tracking of cells used as therapeutic agents.

## Data Availability

See also Supporting Information files.

## Supplementary Materials

The following supporting information can be downloaded at: https://www.mdpi.com/article/10.3390/cells11152323/s1, Figure S1: Dynamic light scattering of GNPs. Multi-angle dynamic light scattering of GNPs after filtration with a 0.2 µm filter, showing the size of the GNPs of 74 nm in diameter; Table S1: Cell loading efficiency determined by ICP-MS. PLX-PAD cells were either incubated with 1 h or 24 with 400 µg/mL GNPs for the low loading and high loading protocol respectively. Afterwards, gold amount in GNP exposed cells was quantified *via* ICP-MS analysis and the percentage of recovered gold as a function of total exposed gold was calculated; Figure S2: Cellular viability of PLX PAD cells after incubation with different concentrations of GNPs. PLX PAD cells were incubated with 25–1200 µg/mL GNPs for 24 h in full DMEM and the percentage of viable cells was determined with an Alamar Blue Assay. Results are shown as mean ± SEM of three independent assays; Figure S3: Hard corona proteins separated by SDS-PAGE and stained by a silver staining. The lanes are labelled according to the incubation time and Lysate corresponds to the cell lysate of PLX PAD cells. Lanes labelled with * are not relevant here; Figure S4: Representative TEM images of PLX PAD cells after first incubation with the low loading protocol followed by a second incubation for 2 h. The scale bars represent 1 µm; Figure S5: Representative TEM images of PLX PAD cells after first incubation with the low loading protocol followed by a second incubation for 6 h. The scale bars represent 1 µm; Figure S6: Representative TEM images of PLX PAD cells after first incubation with the low loading protocol followed by a second incubation for 24 h. The scale bars represent 1 µm; Figure S7: Proteomic analysis of the protein corona on the surface of GNPs after uptake in PLX PAD cells. The heatmap is displaying the combined TOP 10 proteins of each condition (25 proteins in total) identified by LC-MS. The values are reported as the mol% based on all identified proteins in the protein corona; Table S2: List of enriched proteins in the protein corona identified by LC-MS. Displayed are the 1.5-fold enriched proteins compared to the proteins of the lysate; Table S3: List of proteins annotated to the GOTERM “extracellular vesicle” by DAVID. Proteins were identified via LC-MS proteomics workflow and annotated to the corresponding intracellular compartment with DAVID. Displayed are the Uniprot Accession number and the protein name of each protein; Table S4: List of proteins annotated to the GOTERM “cell surface” by DAVID. Proteins were identified via LC-MS and annotated to the corresponding intracellular compartment with DAVID. Displayed are the Uniprot Accession number and the protein name of each protein; Table S5: List of proteins annotated to the secretory vesicle pathway by DAVID. Proteins were identified via LC-MS and annotated to the corresponding intracellular compartment with DAVID. Displayed are the Uniprot Accession number and the protein name of each protein; Table S6: List of proteins annotated to the endoplasmatic reticulum by DAVID. Proteins were identified via LC-MS and annotated to the corresponding intracellular compartment with DAVID. Displayed are the Uniprot Accession number and the protein name of each protein; Table S7: List of proteins annotated to the early endosome pathway by DAVID. Proteins were identified via LC-MS and annotated to the corresponding intracellular compartment with DAVID. Displayed are the Uniprot Accession number and the protein name of each protein; Table S8: List of proteins annotated to the cytoplasmic region by DAVID. Proteins were identified via LC-MS and annotated to the corresponding intracellular compartment with DAVID. Displayed are the Uniprot Accession number and the protein name of each protein; Table S9: List of proteins annotated to the endocytotic vesicle pathway by DAVID. Proteins were identified via LC-MS and annotated to the corresponding intracellular compartment with DAVID. Displayed are the Uniprot Accession number and the protein name of each protein; Table S10: List of proteins annotated to the nucleosome by DAVID. Proteins were identified via LC-MS and annotated to the corresponding intracellular compartment with DAVID. Displayed are the Uniprot Accession number and the protein name of each protein; Table S11: List of proteins in negative control identified by LC-MS. The negative control cells and tubes were treated in the same way as the samples to ensure that only corona proteins are analyzed and to exclude cell contamination.
