# Supplementary material for: Higher Loading of Gold Nanoparticles in PAD Mesenchymal-like Stromal Cells Leads to a Decreased Exocytosis
Source: Cells. 2022 Jul 28;11(15):2323. doi: 10.3390/cells11152323 (PMC9367297; doi:10.3390/cells11152323)
Supplement: Supplementary file 1 [file cells-11-02323-s001.zip › cells-1794838 - supplementary.pdf]

cells

MDPI

Supplementary Information

Higher Loading of Gold Nanoparticles in PAD Mesenchymal-like Stromal Cells Leads to a Decreased Exocytosis

Jennifer Oberländer [1,2], Rafael Ayerbe [3], Joan Cabellos [3], Richard da Costa Marques [1,2], Bin Li [4], Nazende Günday-Türeli [4],Akif Emre Türeli [4], Racheli Ofir [5], Eliran Ish Shalom [5], and Volker Mailänder [1,2,*]

[1] Max-Planck-Institute for Polymer Research, Ackermannweg 10, 55122 Mainz, Germany; oberlaenderj@mpip-mainz.mpg.de (J.O.); dacostamarques@mpip-mainz.mpg.de (R.d.C.M.)
[2] Department of Dermatology, University Medical Center of the Johannes Gutenberg-University Mainz, Langenbeckstraße 1, 55131 Mainz, Germany
[3] LEITAT Technological Center, c/Innovació, 2, 08225 Terrassa, Spain; rayerbe@leitat.org (R.A.); jcabellos@leitat.org (J.C.)
[4] MyBiotech GmbH, Industriestraße 1 B, 66802 Überherrn, Germany; b.li@mybiotech.de (B.L.); n.guenday-tuereli@mybiotech.de (N.G.-T.); e.tuereli@mybiotech.de (A.E.T.)
[5] Pluristem Therapeutics Inc., Matam Park, Building 05, Haifa 3508409, Israel; racheli@pluristem.com (R.O.); ishshalom@pluristem.com (E.I.S.)
* Correspondence: volker.mailaender@mpip-mainz.mpg.de

1. Materials and Methods

Synthesis of GNPs nanoparticles

The nanoparticles were synthesized by heating $HAuCl_4$ solution (50%; 82.345 ml) in ultrapure water (39.78 l) until boiling. Then sodium citrate solution (10%; 803.556 ml) was introduced and the solution was stirred for 10 min at 1200 rpm. Particles were purified and concentrated via cross-flow filtrations with a 10 kDa PES membrane to a final volume of 1193.4 ml. In a second step PEG (PEG7, 50 mg/ml, 198.9 ml) was added and together with the particles stirred for 30 min at 1200 rpm followed by an incubation at 4°C for 24h. Purification was again performed via cross-flow filtration reaching a final volume of 688.5 ml. In the third step, the glucose was attached to the nanoparticles. Therefore, the PEGylated particles were stirred together with 1-Ethyl-3-(3-dimethylaminopropyl)carbodiimid (EDC; 0.2M; 68.85 ml) and N-hydroxysuccinimide (NHS; 0.2M; 344.25 ml) for 30 min at 1200 rpm. Then, the active ester from PEG can react in an amidation reaction with the NH2 groups of the glucosamine (24 mg/ml; 688.5 ml). After the addition of the glucosamine, the solution was stirred again for 30 min at 1200 rpm and then incubated at 4°C for 24 h. Final purification was again performed by cross flow filtration and particles were filtrated through a 0.22 µm filter.

Characterization of GNPs nanoparticles

The size of the GNPs was determined with multi-angle DLS and TEM.

DLS

For multi-angle DLS an ALV spectrometer consisting of a goniometer and an ALV-5004 multiple-tau full digital correlator (320 channels) was used. This allows measurements over an angular range from 30° to 150°. A He-Ne laser (wavelength of 632.8 nm) was used as the light source. Temperature was adjusted through a thermostat from Julabo. Before the measurement, the GNPs were filtrated through a 0.2 µm filter so that no larger particles or dust interfered with the measurement.

TEM

TEM images were taken with a Jeol JEM 1400 at 120 kV to determine the primary size. For the drop casting, undiluted GNPs were added on a 300 mash copper grid coated with a 20-30 nm carbon layer. Excess sample dispersion was blotted with a filter paper[1]. The size was determined using Image J software and counting 100 particles.

ICP-OES

The gold concentration was determined *via* ICP-OES (SpectroGreen, Spectro/Ametek). 10 µl GNPs were diluted in 1 ml aqua regia (3:1 hydrochloric acid: nitric acid) for digestion of nanoparticles. Afterwards, the samples were diluted up to 10 ml with MiliQ water, and the gold concentration was determined with ICP-OES. The calibration curve was prepared by using 0.1, 0.5, 1, 5, and 20 ppm gold standard solution (stock 1000 mg/l Au TraceCERT®, Sigma Aldrich).

Production of PLX PAD cells

Production of PLX PAD cells was performed in a state-of-the-art clean room facility according to GMP regulations. Human placentas were collected from healthy donors and cut into pieces. After enzymatic digestion of the tissue, cells were seeded as 2D cultures in a culture flask followed by 3D cultivation in a bioreactor. Before characterization of the cells, 3D cultures were harvested and cryopreserved in liquid nitrogen. Characterization was performed by staining the cells with MSC-positive and MSC-negative markers and analyzed by flow cytometry[2].

Cell culture

PLX-PAD cells were cultured in Dulbecco's modified eagle medium (DMEM) supplemented with 10% FBS, 100 U ml^{-1} penicillin, 100 mg ml^{-1} streptomycin, and 2 mM glutamine. Viability and count were measured with trypan blue by an automated cell counter (TC10, Bio-Rad). Cells were grown in a humidified incubator at 37°C and 5% CO_2. Cells were either thawed one day before the experiment and seeded at the recommended density for the experiments or cultured in flasks and sub-cultured once a week when they reached around 80% confluence.

Nanoparticle cell uptake analysis by TEM

For the analysis of the uptake of GNPs with the high loading protocol, cells were harvested after the incubation time by scrapping and centrifugation. After washing the cell pellet with phosphate buffer (PB, 0.1M), the cells were stained with OsO4 (1%) for 2 h and washed again with PBS. The cells were dehydrated at 4 °C through a series of acetone concentrations (50%, 70%, 90%, 96%, and 100%), prior to being progressively (25%, 50%, 75%, and 100%) embedded in Epon resin. After resin curing (60 °C, 48 h), sections with a thickness of 50 nm were cut with an ultramicrotome and placed on Formvar carbon-coated Cu grids. Finally, these grids were further contrasted with uranyl acetate and lead citrate. All electron micrographs were obtained with a TEM (Jeol JEM 1010 MT), operating at 80KV. Images were obtained with AnalySIS (SIS, Munster) on a Megaview III CCD camera.

After incubation with the low loading protocol, the GNPs were removed and the sapphire discs with cells were frozen under high pressure (2100 bars) with a high pressure freezing machine (Engineering Office M. Wohlwend GmbH) for a good preservation of the cellular structures. The sapphire discs were locked in a small volume between two specimen carriers and introduced into the specimen pressure chamber with liquid nitrogen as the cooling medium. Additionally, a freeze substitution and resin embedding was added. Therefore, the cryo-fixed samples were dehydrated at -90°C in a freeze substitution machine (EM, AFS 2, Leica Microsystems) by water substitution with an organic solvent (0.2 % osmium tetroxide, 0.1 % uranyl acetate, and 5 % water in acetone). Afterwards, samples were rinsed in acetone at room temperature and with

EPON 812 infiltrated. On the next day, polymerization was performed at 60 °C and then ultrathin sections using a Leica ultramicrotome were produced. All electron micrographs were either obtained with a Jeol JEM 1400 or with a FEI Tecnai F20 TEM at 120 kV and 200 kV respectively.

Protein corona analysis

In-solution digestion, Liquid chromatography-mass spectrometry (LC-MS), and protein identification

Prior to digestion, SDS was removed from the protein mix using Pierce Detergent Removal Spin Columns (Thermo Fisher Scientific). Afterwards, the proteins were precipitated via the ProteoExtract protein precipitation kit (Merck) and solubilized in RapiGest SF (Waters) in ammonium bicarbonate butter (50 mM). The Proteins were reduced with dithiothreitol (5 mM, Sigma-Aldrich) and alkylated with iodoacetamide (15 mM, Sigma-Aldrich) followed by an overnight tryptic digestion at a protein:trypsin ratio of 50:1. To allow absolute protein quantification, samples were diluted with 0.1 % formic acid and spiked with 50 fmol μl^{-1} Hi3 *E.coli.* (Waters), following a published protocol[3].

Peptides were measured using a nanoACQUITY UPLC system coupled to a Synapt G2-Si mass spectrometer. The system was operated in a positive resolution mode, performing data-independent acquisition with additional ion mobility separation (IMS-MS^E) with a mass to charge range of 50–2000 Da, scan time of 1 s, ramped trap collision energy from 20 to 40 V, and data acquisition of 120 min. The samples were injected with a flow rate of 0.3 μl min^{-1} and as a reference Glu-Fibrinopeptide (150 fmol μl^{-1}) and Leu-Enkephalin (200 pg μl^{-1}) were used and injected with a flow rate of 0.5 μl min^{-1}.

Data processing was performed using MassLynx 4.1 and Progenesis GI 2.0 with a reviewed database downloaded from Uniprot. Thresholds for noise reduction were set at 120, 25, and 750 counts for low energy, high energy, and peptide intensity. For protein identification, at least one assigned fragment per peptide, three assigned fragments per protein, and one assigned peptides per protein were required. The TOP3/HI3 approach provided the amount of each protein in fmol[4].

2. Supplementary data

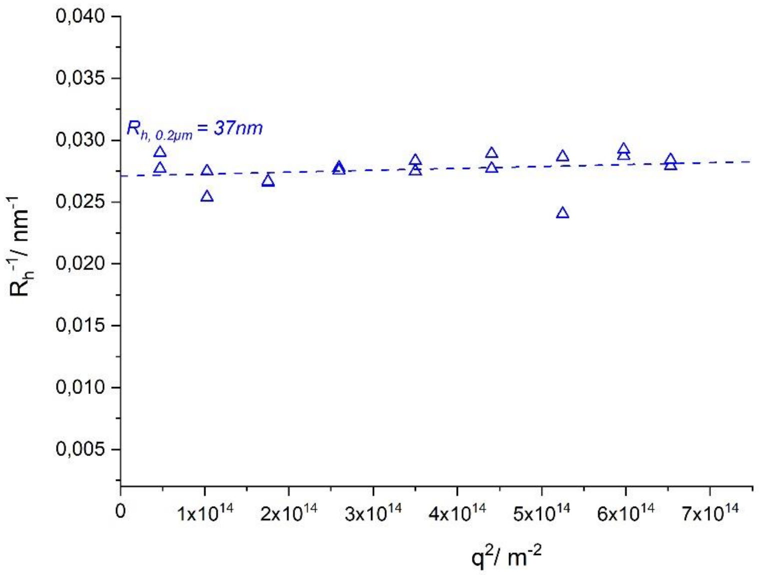

Figure S1. Dynamic light scattering of GNPs. Multi-angle dynamic light scattering of GNPs after filtration with a 0.2 µm filter, showing the size of the GNPs of 74 nm in diameter.

Table S1. Cell loading efficiency determined by ICP-MS. PLX-PAD cells were either incubated with 1 h or 24 with 400 µg/ml GNPs for the low loading and high loading protocol respectively. Afterwards, gold amount in GNP exposed cells was quantified *via* ICP-MS analysis and the percentage of recovered gold as a function of total exposed gold was calculated.

	Low Loading		High Loading	
	Recovery of Au vs Total Au (%)	SD	Recovery of Au vs Total Au (%)	SD
Supernatant 1	86.5	3.1	66.6	9.2
Supernatant 2 (1st wash)	7.2	1.7	4.3	0.8
Supernatant 3 (2nd wash)	n.a	n.a	1.0	0.2
Cells	6.4	0.3	27.9	7.7
Total Au recovered (%)	104.9	5.4	105.0	3.8

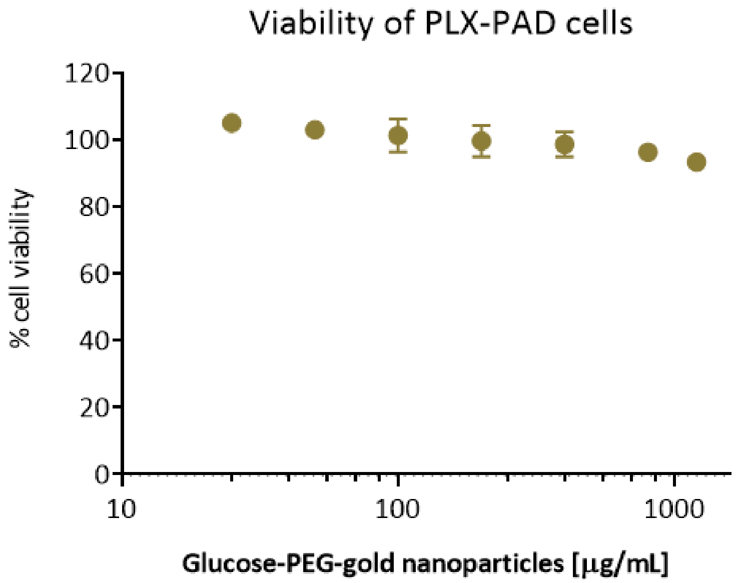

Figure S2. Cellular viability of PLX PAD cells after incubation with different concentrations of GNPs. PLX PAD cells were incubated with 25-1200 µg/ml GNPs for 24 h in full DMEM and the percentage of viable cells was determined with an Alamar Blue Assay. Results are shown as mean ± SEM of three independent assays.

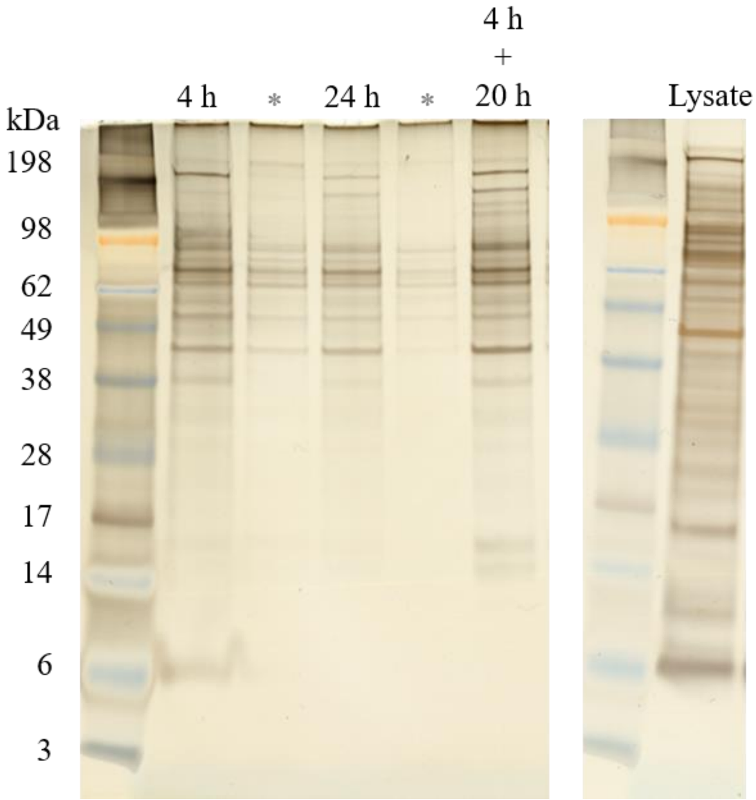

Figure S3. Hard corona proteins separated by SDS-PAGE and stained by a silver staining. The lanes are labelled according to the incubation time and Lysate corresponds to the cell lysate of PLX PAD cells. Lanes labelled with * are not relevant here.

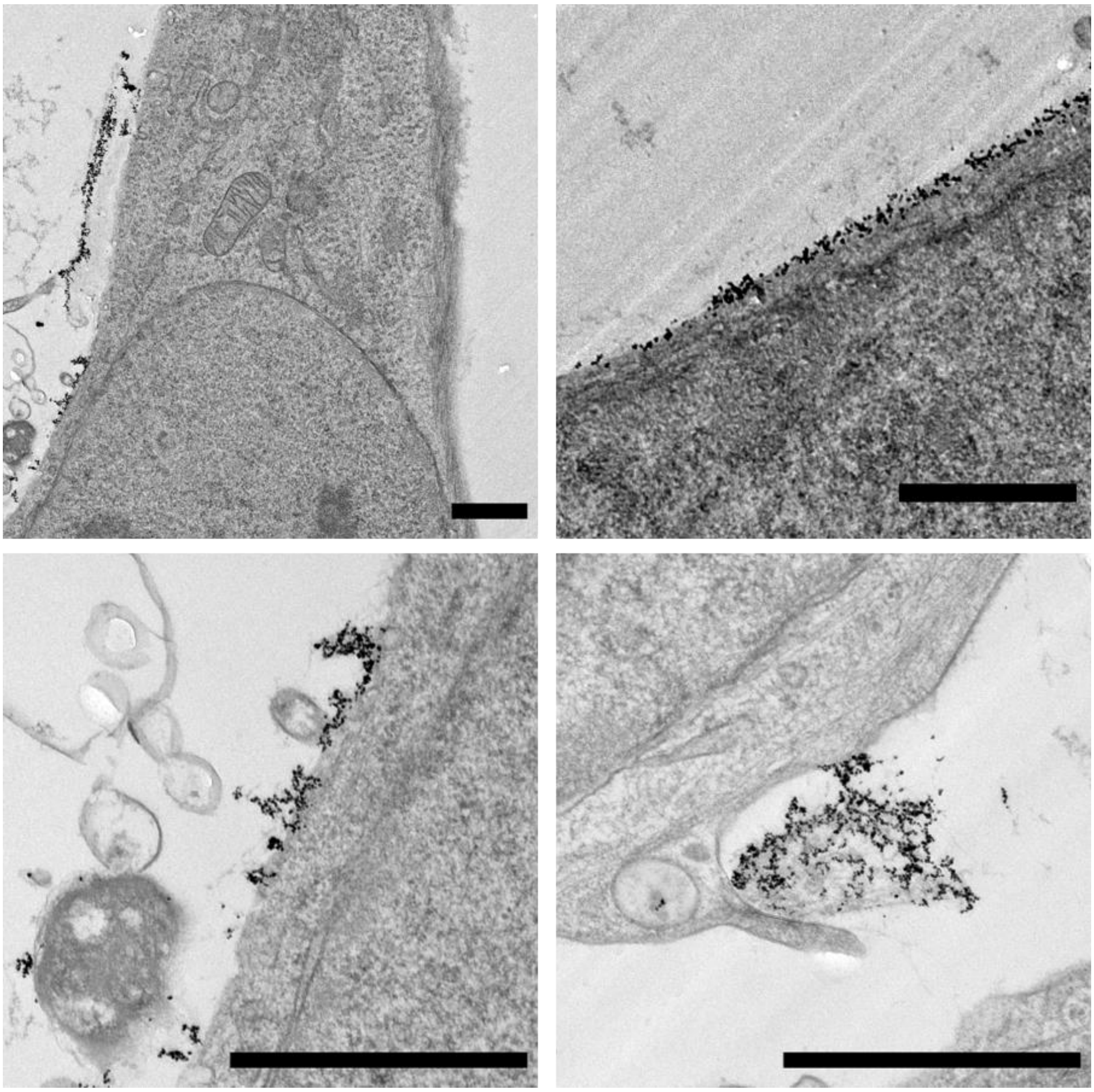

Figure S4. Representative TEM images of PLX PAD cells after first incubation with the low loading protocol followed by a second incubation for 2 h. The scale bars represent 1 µm.

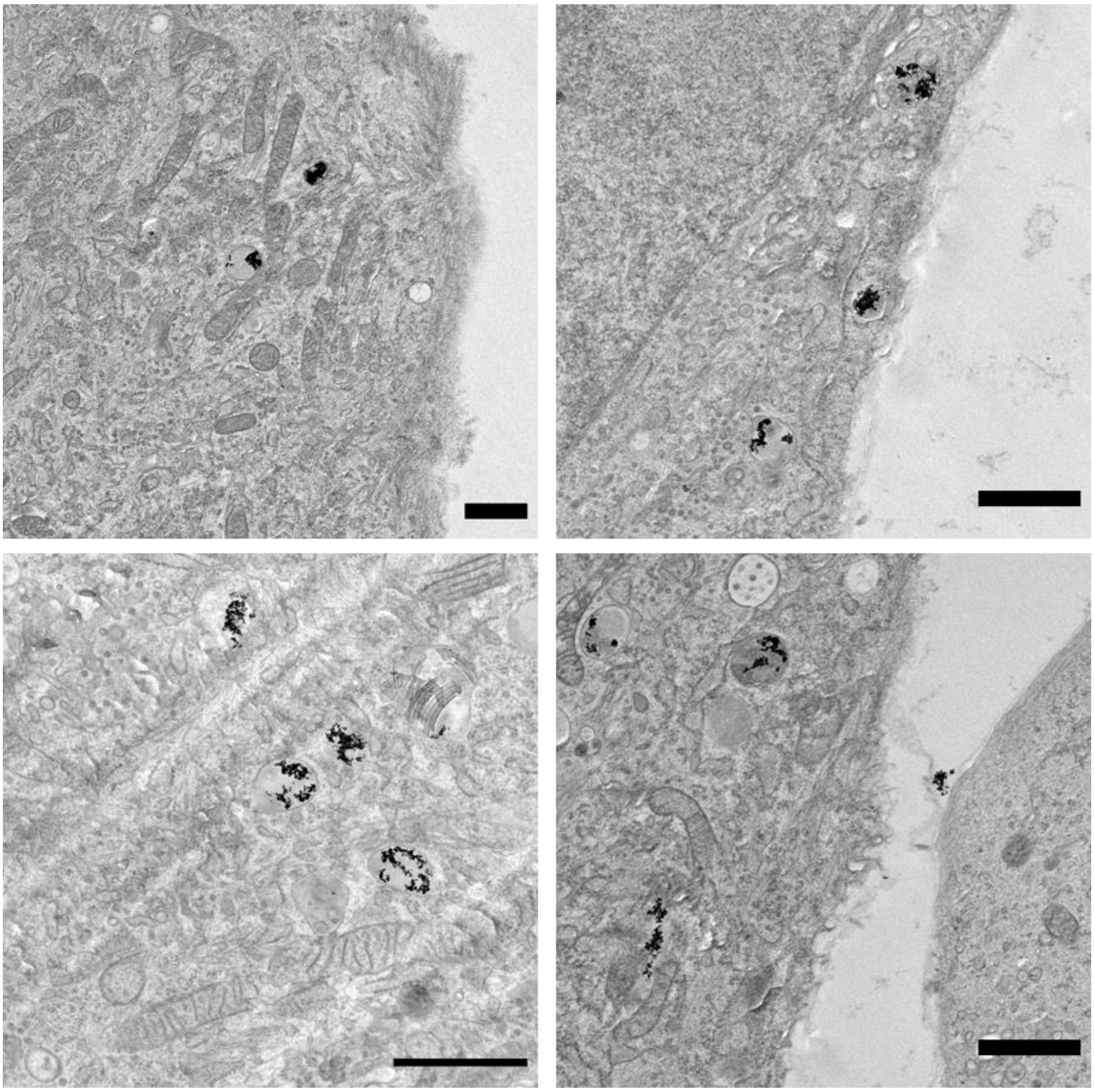

Figure S5. Representative TEM images of PLX PAD cells after first incubation with the low loading protocol followed by a second incubation for 6 h. The scale bars represent 1 μm.

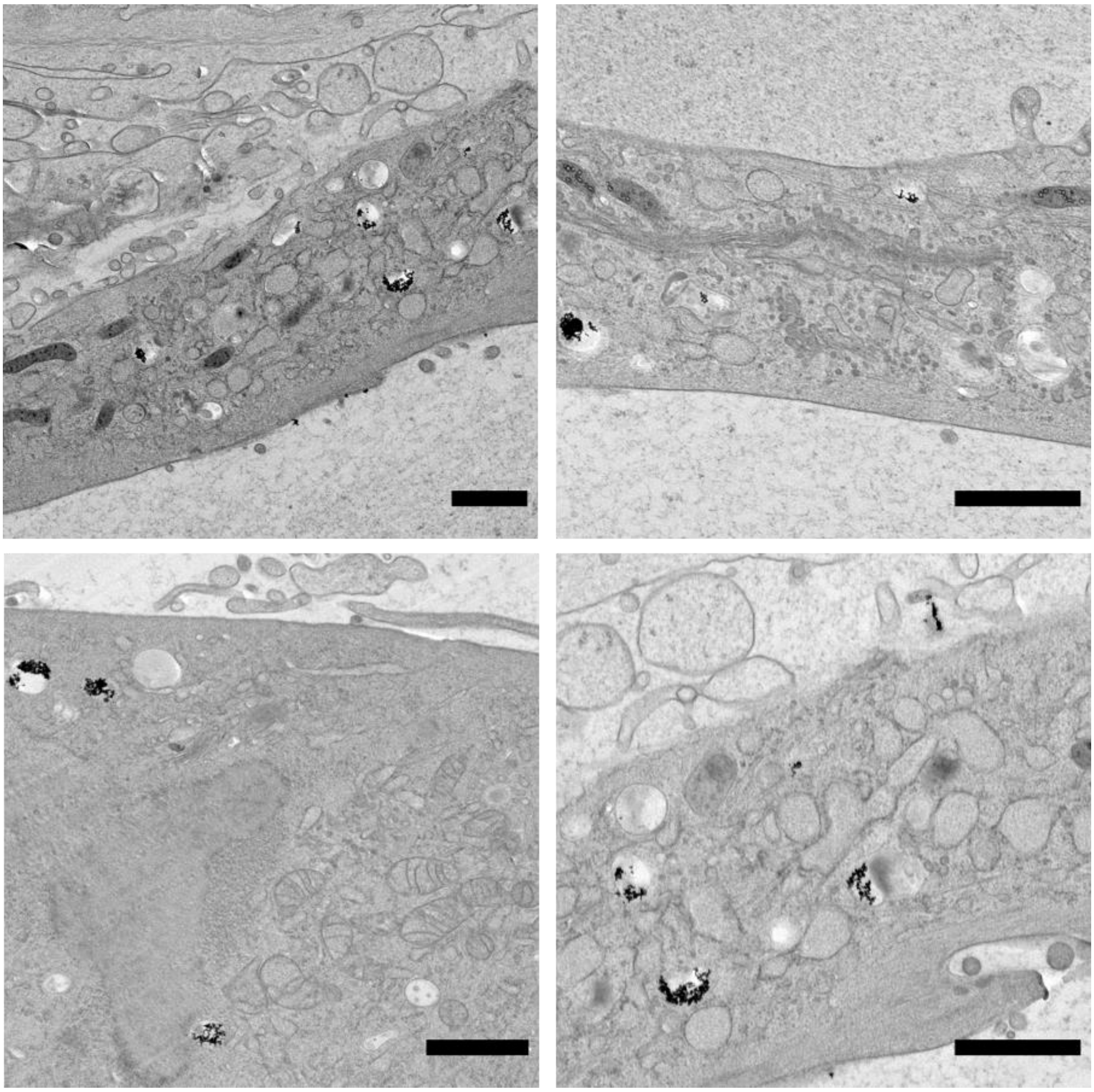

Figure S6. Representative TEM images of PLX PAD cells after first incubation with the low loading protocol followed by a second incubation for 24 h. The scale bars represent 1 μm.

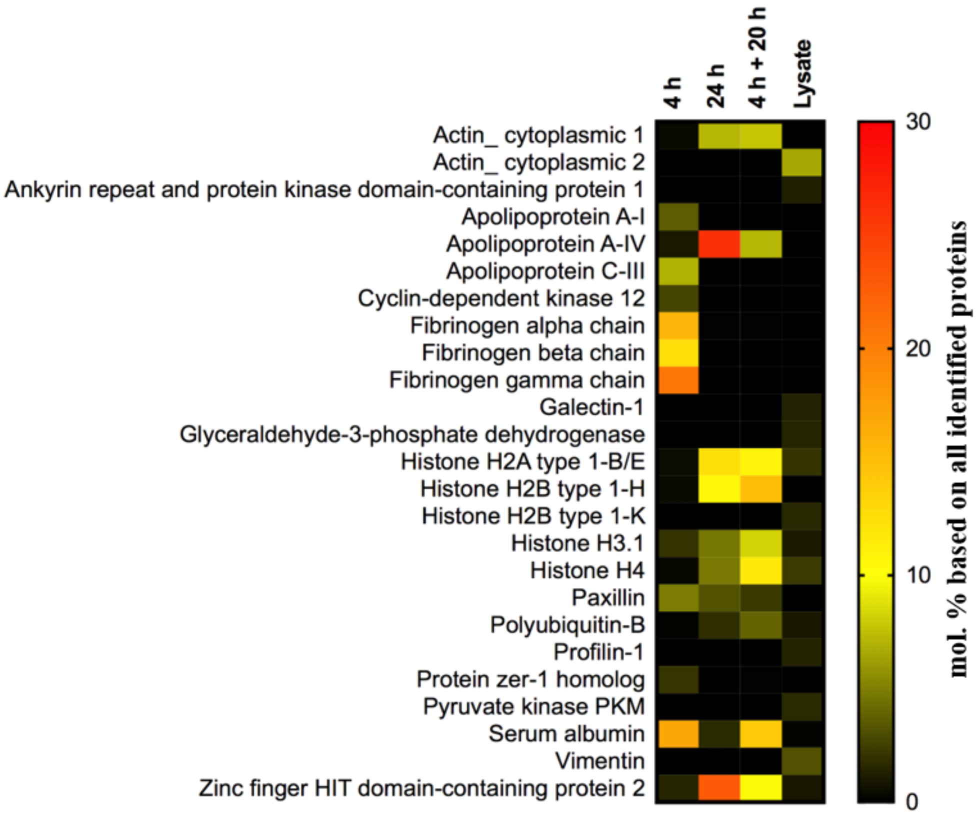

Figure S7. Proteomic analysis of the protein corona on the surface of GNPs after uptake in PLX PAD cells. The heatmap is displaying the combined TOP 10 proteins of each condition (25 proteins in total) identified by LC-MS. The values are reported as the mol% based on all identified proteins in the protein corona.

Table S2. List of enriched proteins in the protein corona identified by LC-MS. Displayed are the 1.5-fold enriched proteins compared to the proteins of the lysate.

Uniprot Accession	Protein Name
P62249	40S ribosomal protein S16
P62851	40S ribosomal protein S25
P60709	Actin_ cytoplasmic 1
P12235	ADP/ATP translocase 1
P05141	ADP/ATP translocase 2
P01009	Alpha-1-antitrypsin
P01023	Alpha-2-macroglobulin
P02647	Apolipoprotein A-I
P06727	Apolipoprotein A-IV
P02655	Apolipoprotein C-II
P02656	Apolipoprotein C-III
P02649	Apolipoprotein E
O43150	Arf-GAP with SH3 domain_ ANK repeat and PH domain-containing protein 2
P25705	ATP synthase subunit alpha_ mitochondrial
P25098	Beta-adrenergic receptor kinase 1
P10909	Clusterin
P01024	Complement C3
Q9NYV4	Cyclin-dependent kinase 12
Q07065	Cytoskeleton-associated protein 4
P04843	Dolichyl-diphosphooligosaccharide--protein glycosyltransferase subunit 1
P02671	Fibrinogen alpha chain
P02675	Fibrinogen beta chain
P02679	Fibrinogen gamma chain
P00738	Haptoglobin
Q8WYB5	Histone acetyltransferase KAT6B
P10412	Histone H1.4
P16401	Histone H1.5
P04908	Histone H2A type 1-B/E
Q93079	Histone H2B type 1-H
P68431	Histone H3.1
P62805	Histone H4
Q14520	Hyaluronan-binding protein 2
P01876	Immunoglobulin heavy constant alpha 1
P01857	Immunoglobulin heavy constant gamma 1
P01861	Immunoglobulin heavy constant gamma 4
P01871	Immunoglobulin heavy constant mu
P01834	Immunoglobulin kappa constant
P0DOY2	Immunoglobulin lambda constant 2
Q5T7N2	LINE-1 type transposase domain-containing protein 1
P49023	Paxillin
P0CG47	Polyubiquitin-B
Q13045	Protein flightless-1 homolog
P61619	Protein transport protein Sec61 subunit alpha isoform 1
Q7Z7L7	Protein zer-1 homolog
P51153	Ras-related protein Rab-13
P02787	Serotransferrin
P02768	Serum albumin
P0DPH7	Tubulin alpha-3C chain
P04004	Vitronectin
Q9UHR6	Zinc finger HIT domain-containing protein 2
P21506	Zinc finger protein 10

Table S3. List of proteins annotated to the GOTERM "extracellular vesicle" by DAVID. Proteins were identified *via* LC-MS proteomics workflow and annotated to the corresponding intracellular compartment with DAVID. Displayed are the Uniprot Accession number and the protein name of each protein.

**Extracellular Vesicle**	
**Uniprot Accession**	**Protein Name**
P62249	40S ribosomal protein S16
P62851	40S ribosomal protein S25
P60709	Actin_ cytoplasmic 1
P05141	ADP/ATP translocase 2
P01009	Alpha-1-antitrypsin
P01023	Alpha-2-macroglobulin
P02647	Apolipoprotein A-I
P06727	Apolipoprotein A-IV
P02655	Apolipoprotein C-II
P02656	Apolipoprotein C-III
P02649	Apolipoprotein E
P25705	ATP synthase subunit alpha_ mitochondrial
P10909	Clusterin
P01024	Complement C3
Q07065	Cytoskeleton-associated protein 4
P02671	Fibrinogen alpha chain
P02675	Fibrinogen beta chain
P02679	Fibrinogen gamma chain
P00738	Haptoglobin
P10412	Histone H1.4
P16401	Histone H1.5
P04908	Histone H2A type 1-B/E
Q93079	Histone H2B type 1-H
P68431	Histone H3.1
P62805	Histone H4
P01876	Immunoglobulin heavy constant alpha 1
P01857	Immunoglobulin heavy constant gamma 1
P01861	Immunoglobulin heavy constant gamma 4
P01871	Immunoglobulin heavy constant mu
P01834	Immunoglobulin kappa constant
P0DOY2	Immunoglobulin lambda constant 2
P0CG47	Polyubiquitin-B
P51153	Ras-related protein Rab-13
P02787	Serotransferrin
P02768	Serum albumin
P04004	Vitronectin

Table S4. List of proteins annotated to the GOTERM "cell surface" by DAVID. Proteins were identified *via* LC-MS and annotated to the corresponding intracellular compartment with DAVID. Displayed are the Uniprot Accession number and the protein name of each protein.

Cell Surface	
**Uniprot Accession**	**Protein Name**
P02647	Apolipoprotein A-I
P06727	Apolipoprotein A-IV
P10909	Clusterin
P02671	Fibrinogen alpha chain
P02675	Fibrinogen beta chain
P02679	Fibrinogen gamma chain
P01876	Immunoglobulin heavy constant alpha 1
P01857	Immunoglobulin heavy constant gamma 1
P01861	Immunoglobulin heavy constant gamma 4
P01871	Immunoglobulin heavy constant mu
P01834	Immunoglobulin kappa constant
P0DOY2	Immunoglobulin lambda constant 2
P02787	Serotransferrin

Table S5. List of proteins annotated to the secretory vesicle pathway by DAVID. Proteins were identified *via* LC-MS and annotated to the corresponding intracellular compartment with DAVID. Displayed are the Uniprot Accession number and the protein name of each protein.

Secretory Vesicle	
**Uniprot Accession**	**Protein Name**
P01009	Alpha-1-antitrypsin
P01023	Alpha-2-macroglobulin
P02647	Apolipoprotein A-I
P10909	Clusterin
Q07065	Cytoskeleton-associated protein 4
P02671	Fibrinogen alpha chain
P02675	Fibrinogen beta chain
P02679	Fibrinogen gamma chain
P51153	Ras-related protein Rab-13
P02787	Serotransferrin
P02768	Serum albumin

Table S6. List of proteins annotated to the endoplasmatic reticulum by DAVID. Proteins were identified *via* LC-MS and annotated to the corresponding intracellular compartment with DAVID. Displayed are the Uniprot Accession number and the protein name of each protein.

Endoplasmatic Reticulum	
**Uniprot Accession**	**Protein Name**
P01009	Alpha-1-antitrypsin
P02647	Apolipoprotein A-I
P06727	Apolipoprotein A-IV
P02649	Apolipoprotein E
P10909	Clusterin
Q07065	Cytoskeleton-associated protein 4
P04843	Dolichyl-diphosphooligosaccharide--protein glycosyltransferase subunit 1
P61619	Protein transport protein Sec61 subunit alpha isoform 1
P02768	Serum albumin
P04004	Vitronectin

Table S7. List of proteins annotated to the early endosome pathway by DAVID. Proteins were identified *via* LC-MS and annotated to the corresponding intracellular compartment with DAVID. Displayed are the Uniprot Accession number and the protein name of each protein.

Early Endosome	
**Uniprot Accession**	**Protein Name**
P02647	Apolipoprotein A-I
P06727	Apolipoprotein A-IV
P02655	Apolipoprotein C-II
P02656	Apolipoprotein C-III
P02649	Apolipoprotein E
P02787	Serotransferrin

Table S8. List of proteins annotated to the cytoplasmic region by DAVID. Proteins were identified *via* LC-MS and annotated to the corresponding intracellular compartment with DAVID. Displayed are the Uniprot Accession number and the pro-tein name of each protein.

Cytoplasmatic Region	
**Uniprot Accession**	**Protein Name**
P60709	Actin_ cytoplasmic 1
P02671	Fibrinogen alpha chain
P02675	Fibrinogen beta chain
P02679	Fibrinogen gamma chain
P49023	Paxillin

Table S9. List of proteins annotated to the endocytotic vesicle pathway by DAVID. Proteins were identified *via* LC-MS and annotated to the corresponding intracellular compartment with DAVID. Displayed are the Uniprot Accession number and the protein name of each protein.

Endocytotic Vesicle	
**Uniprot Accession**	**Protein Name**
P02647	Apolipoprotein A-I
P02649	Apolipoprotein E
P00738	Haptoglobin
P0CG47	Polyubiquitin-B
P51153	Ras-related protein Rab-13
P02787	Serotransferrin

Table S10. List of proteins annotated to the nucleosome by DAVID. Proteins were identified *via* LC-MS and annotated to the corresponding intracellular compartment with DAVID. Displayed are the Uniprot Accession number and the protein name of each protein.

Nucleosome	
**Uniprot Accession**	**Protein Name**
Q8WYB5	Histone acetyltransferase KAT6B
P10412	Histone H1.4
P16401	Histone H1.5
P04908	Histone H2A type 1-B/E
Q93079	Histone H2B type 1-H
P68431	Histone H3.1
P62805	Histone H4

Table S11. List of proteins in negative control identified by LC-MS. The negative control cells and tubes were treated in the same way as the samples to ensure that only corona proteins are analyzed and to exclude cell contamination.

Negative Control		
**Uniprot Accession**	**Protein Name**	**Mol %**
P62736	Actin_ aortic smooth muscle	4,54
P60709	Actin_ cytoplasmic 1	6,94
P05141	ADP/ATP translocase 2	0,36
P12236	ADP/ATP translocase 3	0,41
P25705	ATP synthase subunit alpha_ mitochondrial	0,48
P06576	ATP synthase subunit beta_ mitochondrial	0,29
P00403	Cytochrome c oxidase subunit 2	0,30
Q07065	Cytoskeleton-associated protein 4	0,16
P10412	Histone H1.4	1,55
P16401	Histone H1.5	0,92
P04908	Histone H2A type 1-B/E	13,43
P0C0S5	Histone H2A.Z	0,41
O60814	Histone H2B type 1-K	10,10
P68431	Histone H3.1	5,87
P62805	Histone H4	14,02
P60660	Myosin light polypeptide 6	0,31
O14950	Myosin regulatory light chain 12B	0,22
P35579	Myosin-9	1,35
Q00325	Phosphate carrier protein_ mitochondrial	0,61
Q96DU9	Polyadenylate-binding protein 5	1,45
P0CG47	Polyubiquitin-B	1,76
P02545	Prelamin-A/C	0,33
P35232	Prohibitin	0,17
Q6UXU0	Putative uncharacterized protein	0,26
P06753	Tropomyosin alpha-3 chain	0,57
Q8NEP4	Uncharacterized protein C17orf47	23,96
O95399	Urotensin-2	0,06
P08670	Vimentin	0,30
Q5T200	Zinc finger CCCH domain-containing protein 13	1,35
Q9UHR6	Zinc finger HIT domain-containing protein 2	7,54

References

1. Renz, P.; Kokkinopoulou, M.; Landfester, K.; Lieberwirth, I., Imaging of polymeric nanoparticles: hard challenge for soft objects. *Macromolecular Chemistry and Physics* **2016,** *217* (17), 1879-1885.
2. Winkler, T.; Perka, C.; von Roth, P.; Agres, A. N.; Plage, H.; Preininger, B.; Pumberger, M.; Geissler, S.; Hagai, E. L.; Ofir, R.; Pinzur, L.; Eyal, E.; Stoltenburg-Didinger, G.; Meisel, C.; Consentius, C.; Streitz, M.; Reinke, P.; Duda, G. N.; Volk, H. D., Immunomodulatory placental-expanded, mesenchymal stromal cells improve muscle function following hip arthroplasty. *J Cachexia Sarcopenia Muscle* **2018,** *9* (5), 880-897.
3. Bradshaw, R. A.; Burlingame, A. L.; Carr, S.; Aebersold, R., Reporting protein identification data: the next generation of guidelines. *Molecular & Cellular Proteomics* **2006,** *5* (5), 787-788.
4. Silva, J. C.; Gorenstein, M. V.; Li, G. Z.; Vissers, J. P.; Geromanos, S. J., Absolute quantification of proteins by LCMSE: a virtue of parallel MS acquisition. *Mol. Cell. Proteomics* **2006,** *5* (1), 144-56.
